# Effects of Curcumin on Vessel Formation Insight into the Pro- and Antiangiogenesis of Curcumin

**DOI:** 10.1155/2019/1390795

**Published:** 2019-06-19

**Authors:** Ting-ye Wang, Jia-xu Chen

**Affiliations:** School of Traditional Chinese Medicine, Beijing University of Chinese Medicine, Beijing 100029, China

## Abstract

Curcumin is a compound extracted from the* Curcuma longa L,* which possesses a wide range of pharmacological effects. However, few studies have collected scientific evidence on its dual effect on angiogenesis. The present review gathered the fragmented information available in the literature to discuss the dual effect and possible mechanisms of curcumin on angiogenesis. Available information concerning the effect of curcumin on angiogenesis is compiled from scientific databases, including PubMed and Web of Science using the key term (curcumin and angiogenesis). The results were reviewed to identify relevant articles. Related literature demonstrated that curcumin has antiangiogenesis effect via regulating multiple factors, including proangiogenesis factor VEGF, MMPs, and FGF, both* in vivo* and* in vitro*, and could promote angiogenesis under certain circumstances via these factors. This paper provided a short review on bidirectional action of curcumin, which should be useful for further study and application of this compound that require further studies.

## 1. Introduction

**Figure 1:**
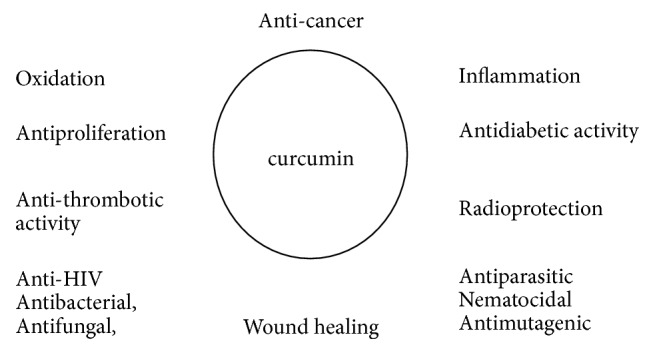
Biological effect of curcumin.

Curcumin, one of the most promising natural compounds, is the major polyphenol compound found in* Curcuma longa L* with the symmetric chemical structure [1, 2]. Since its first isolation in impure in 1815 by Vogel and Pelletier [3], Lampe et al. confirmed its chemical structure and synthesis in 1910 and 1913, respectively [4], and identified the use of curcumin in human diseases in 1937 for the first time [5]; extensive studies over the last half century have clearly confirmed the pharmacological and biological effects of curcumin including antiproliferation, anti-inflammatory, antioxidant, anti-HIV, antibacterial, antifungal, nematocidal, antispasmodic, antiparasitic, antimutagenic, antidiabetic, antifibrinolytic, antithrombotic, radioprotective, and anticarcinogenic activity as well as wound healing, lipid lowering, and immunomodulating (Figure 1) [2, 6–11]. Preclinical and clinical researches demonstrated that curcumin could be utilized in the treatment of cancer, diabetes, and other diseases [12]. Cancer is one of the research hotspots in recent years; as a natural compound with no toxicity and promising feature on tumor therapy, attention have been paid on curcumin.

Angiogenesis is the process of new vessel formation and hallmark of tumor progression [13], which is crucial for tumor growth and expansion [14]. It was reported that solid tumor cannot grow well without inducing blood supply [15]. As a result of the hotspot of cancer therapy, the anticarcinogenic effect of curcumin has been investigated systematically, where angiogenesis plays an important role. Studies have found that the anticancer effect of curcumin is achieved by inhibiting angiogenesis partly [16–19]; naturally, effects of curcumin on angiogenesis draw the attention of researchers. At the same time, angiogenesis represents a critical determinant in wound repair where curcumin plays a role because new blood vessels act as a route for delivering oxygen and nutrients to cells at the wound points [20]; effects of curcumin on promoting angiogenesis in wound healing process have been studied. Altogether, curcumin has bidirectional action on angiogenesis.

This article is aimed at reviewing the bidirectional action of curcumin and curcuminoids as well as synthetic curcumin analog on angiogenesis based on current research findings, focusing on regulation of curcumin on proangiogenesis factors in antiangiogenesis and proangiogenesis process; some of the mechanisms were summarized and discussed. Furthermore, this article provided some information and insights which could be interesting to researchers in related areas.

## 2. Traditional Uses of Curcumin

**Figure 2:**
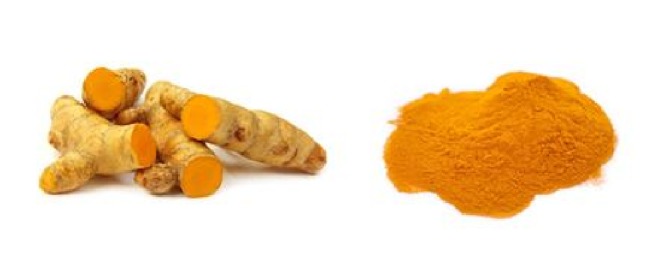
Curcuma rhizome and curcumin powder.

Curcumin (Figure 2) is a member of the ginger family and is prescribed abundantly for ailments in both traditional Chinese and Indian medicine [21]. In China,* Curcuma longa L* has been used as a commonly used traditional Chinese medicine for thousands of years with the effect of activating qi flowing, removing blood stasis, benefiting menstruation, and relieving pain. In India, turmeric is commonly used in the Indian subcontinent as a spice, concurrently used for health care including various respiratory diseases [22, 23]. Besides, turmeric preparations could be applied to treat fresh wounds and anticancer effect of turmeric was also documented in Indian medical literature [24].

## 3. Chemistry of Curcumin

**Figure 3:**
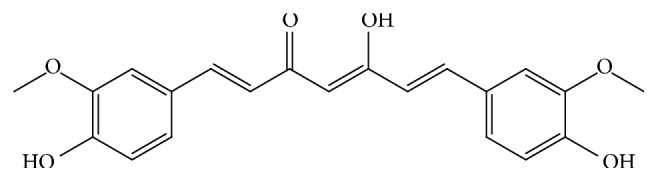
Chemical structure of curcumin.

Chemically, curcumin is a bis-*α*,*β*-unsaturated *β*-diketone [4] (Figure 3). Based on its *β*-diketone moiety, curcumin exists in keto-enol tautomers, and this tautomerism favors interaction and binding with a wide range of enzymes [25]. Some researchers have reported that the potency for the suppression of nuclear factor-kappaB (NF-*κ*B) differ between curcuminoids, suggesting that the methoxy groups on the phenyl rings in curcumin are important to have health effects [26]. In the same time, the 4,4′-free phenolic groups is found to be another important groups in curcumin structure, studies have confirmed 4,4′-free phenolic groups to be associated with curcumin activities [27, 28], and these findings draw attention to the researches on the synthetic analogs of curcumin.

## 4. Bioactivity of Curcumin

Studies over the past thirty years have revealed that low bioavailability of curcumin mainly due to poor absorption and rapid metabolism [29]; metabolism has been considered to be the main reasons of poor bioavailability [30]. Because of the low bioavailability, innovative methods of increasing solution of curcumin have been used, resulting in the highlight on curcumin nanoformulations. During the last decades, various types of nanocarriers of curcumin have been investigated to improve the bioavailability of curcumin and some systems have reached clinical evaluations and applications [31–33]; these achievements can facilitate the better use of curcumin.

## 5. Curcumin and Angiogenesis

In adult, angiogenesis is required for wound healing and female reproductive organs actions [34]. Generally, the angiogenesis process is activated by growth factors such as basic fibroblast growth factor (bFGF), vascular endothelia growth factor (VEGF), or placental growth factor [35]. In adults, the formation and growth of new blood vessels are tightly controlled. These processes are triggered only under strictly defined conditions like wound healing. The function of strict system regulation and balance is very important for the body, because both excessive formation of blood vessels and underdevelopment of blood vessels could lead to serious diseases [36], such as neurological disease and tumor. There are three stages of angiogenesis: the first stage: some endothelial cells, namely, “tip cells” inside the capillary that react to the angiogenesis factor VEGF-A, are selected as the starting point to begin angiogenesis expansion. The second stage: the tip cells only respond to VEGF-A through guided migration; in the sprout stalks, the proliferative response to VEGF-A occurs. Both of these two cellular responses are mediated by the activation of VEGF-A on VEGFR-2 [37]. The third stage: maturation of newly formed vessels consists of endothelial proliferation inhibition, new capillaries migration, and new vascular tubes that already existing stabilization [38, 39]. During the process of angiogenesis, various signaling pathways, related factors, and receptors are involved. VEGF is part of the most important ligand among them. Others as epidermal growth factor (EGF), transforming growth factors (TGF), fibroblast growth factors (FGF), angiopoietin-1 and 2, and matrix metalloproteinases (MMPs) also play a role in the process of angiogenesis [34, 40].

### 5.1. Antiangiogenesis Effects of Curcumin

It was showed that pathological angiogenesis is a mark of cancer and several ischaemic and inflammatory disease [41]. During the past years, great progress has been made in comprehending the mechanism of angiogenesis in different pathophysiological conditions, the antiangiogenesis effect of curcumin is among them. Angiogenesis inhibitors can fall into two categories. The first class, also known as the direct angiogenesis inhibitors, refers to those inhibitors which are relatively sensitive for endothelial cells than tumor cells. The additional class, on the other hand, is the indirect inhibitors, which may have no direct effects on endothelial cells, but may regulate angiogenesis via downregulating an angiogenesis stimulator [42]. Curcumin is a direct inhibitor of angiogenesis; in the meantime, it can also downregulate several proangiogenesis factors [43]. Curcumin affects the whole process of angiogenesis through downregulating transcription factors such as NF-*κ*B and proangiogenesis factors such as VEGF, bFGF, and MMPs [43], all of which are closely and directly linked with tumorigenesis, involving in the complicated regulating process of curcumin.

#### 5.1.1. Fibroblast Growth Factor

bFGF was isolated from bovine pituitary known as highly angiogenic and widely expressed in normal and malignant tissues. bFGF induces angiogenesis as a result of its effects on smooth muscle cells and endothelial cells, as well as its role as a chemoattractant and aid in the proliferation of fibroblasts and epithelial cells. bFGF is expressed in vascular endothelium during tumor neovascularization and antiproliferative diseases. Besides their angiogenic activity, the FGFs are critical for wound healing [44]. Because bFGF is essential preconditions for initiation angiogenesis process, researches focusing on regulation of curcumin on bFGF have been carried out. Results showed that curcumin could inhibit bFGF and FGF-induced angiogenesis in vivo [42, 45]. In an investigation, it was reported that curcumin and its derivatives significantly inhibit corneal neovascularization induced by basic fibroblast growth factor [42]. And a previous study revealed that curcumin could inhibit FGF-induced neovascularization [46], indicating FGFs play an important role in inhibiting angiogenesis by curcumin.

#### 5.1.2. Matrix Metalloproteinases

MMPs have an expanded role in angiogenesis, as they are essential for the creation and maintenance of supporting growth and angiogenesis of tumor [47]. MMPs released by endothelial cells represent a key process in neovascularization. Within all MMPs, MMP-9 plays a regulatory role in angiogenesis not only through proteolytic activity, but also through other downstream angiogenesis factors; study revealed that MMP-9 participated in the angiogenesis of tumors by increasing the effectiveness of VEGF, an important angiogenesis inducer in malignant tumor, indicating that the MMP-9 is a part of the angiogenic regulation [48].

Curcumin shows antiangiogenesis effect via meditating MMP level. A significant decrease of MMP-2 and MMP-9 levels has been identified in the prostate and breast cancer cells treated with curcumin [49]. Experiments concerning curcumin inhibit angiogenesis in glioblastoma xenografts have been carried out and the results showed that the antiangiogenesis activity of curcumin is at least partly via MMP-9 both* in vivo *and* vitro *[50]. These results demonstrated that the inhibition of MMP-9 is one of the major causes of angiogenesis inhibition by demethoxycurcumin [51]. It was observed that curcumin and its synthetic analogs downregulate the expression of genes responsible for angiogenesis and other angiogenesis factors such as VEGF and MMP-9 [52]

#### 5.1.3. Vascular Endothelial Growth Factor

VEGF as the best known angiogenesis factor present was first discovered in 1986 by Senger and his colleagues [53], has been taken as the most critical factor in angiogenesis regulation processes, and is known to be required for normal as well as pathological angiogenesis in many tissues, having a key role in cancer biology and being involved in neovascularization [54]. There are three known VEGF receptors that are VEGFR-1 and VEGFR-2, expressed on vascular endothelial cells and as well as VEGFR-3; VEGF directly initiate an angiogenesis process via binding to its receptors on vascular endothelial cells [55, 56].

Curcumin shows antiangiogenesis effect primarily in tumor both* in vitro* and* in vivo*. Curcumin could reduce the suppression of VEGF in an* in vitro* model of endometriosis [57] and block angiogenesis induced by hypoxia* in vitro* and downregulated VEGF expression [58].* In vivo*, study found that curcumin inhibited angiogenesis through reducing microvessel density in Ehrlich ascites carcinoma-bearing mice; possible mechanism was proved to be inhibition of VEGF and VEGFR2 [59] and is able to inhibit tumor angiogenesis by the reduction of proangiogenesis factor VEGF in the Xenograft model of breast cancer [60]. Other than cancer, curcumin is seen as an angiogenesis inhibitor by downregulating VEGF in corneal diseases, diabetic retinopathy, diabetic nephropathy, and ectopic endometrium [61–65]. Other than tumor, curcumin effectively prevented the angiogenesis response in aortic ring models in both the diabetic and nondiabetic environment where VEGF level decreased [66]. A previous clinical trial demonstrated that curcumin could reduce VEGF level where VEGF overexpression and subsequent vasculogenesis and angiogenesis are implicated in the development of several pathological processes [67]. Another phase I trial revealed that curcumin/docetaxel combination could significantly decrease VEGF levels after three cycles of treatment [68]. Expect for curcumin, liposome curcumin and curcumin nanoparticle also showed antiangiogenesis effect by inhibiting VEGF [69, 70]

#### 5.1.4. VEGF-Related Mediating Factors

COX-2 is an inducible enzyme that is upregulated responsing growth factors stimuli as VEGF [71]. Numerous reports showed that one of the mechanisms of COX-2 participates in tumorigenesis is to induce angiogenesis [72]. Finding brought us the idea that the overexpression of COX-2 may be functionally significant for the early stage of tumor angiogenesis. The effects of COX-2 on tumor angiogenesis might be mediated by the upregulation of angiogenesis factors like VEGF expression. Other results showed the significant correlation between VEGF expression, COX-2 expression, and mast cell density (MCD), indicating COX-2 and MCD may contribute to tumor angiogenesis by regulating the production of VEGF [73]. All together showed that COX-2 induce angiogenesis is closely related to VEGF.* In vitro*, curcumin could inhibit hepatocellular carcinoma cells angiogenesis through reducing the expression of COX-2 and VEGF [74]. Besides, curcumin inhibits angiogenesis in microvascular endothelial cell via suppressing COX-2 expression [75]. Curcumin analogs EF31 and UBS109 also induced the downregulation of COX-2 and VEGF in pancreatic cancer [76]. These findings indicated that COX-2 plays an important in inhibiting angiogenesis by curcumin.

NF-*κ*B could affect the angiogenesis through regulating angiogenesis factors including MMPs and VEGF, and the production of these angiogenesis factors is regulated by NF-*κ*B activation [77]. Activation of NF-*κ*B and its gene products (e.g., VEGF, MMP-2, MMP-9, and COX-2) can be inhibited by curcumin both* in vitro* and* in vivo*, which has a significant role in angiogenesis [78, 79]; these findings might help in the curcumin inhibiting angiogenesis in ovarian carcinoma* in vitro *and* vivo*; results showed decreasing of microvessel density, which is regulated by targeting the nuclear factor-*κ*B pathway [80]. Curcumin nanoparticle could also prevent corneal neovascularization by inhibiting NF-*κ*B in corneal cells induced by lipopolysaccharide [69]. These studies further emphasize NF-*κ*B activation in mediating angiogenesis and curcumin could downregulate NF-*κ*B to inhibit angiogenesis.

### 5.2. Proangiogenesis Effects of Curcumin

Despite the antiangiogenesis of curcumin has been discussed a lot; it is found that curcumin possess a proangiogenesis effect. Study found out that curcumin pretreatment augmented adipose derived stem cells production of VEGF, which contributed to neovessels formation and improving cells survival [81]. A previous study found that curcumin could increase MMP-2, transforming growth factor (TGF)-beta, and VEGF expression, which are proangiogenesis factors and accelerate angiogenesis in an indomethacin-induced model [82]. Curcumin enhanced endothelial progenitor cells (EPCs) function, namely, angiogenesis, migration, and proliferation ability, and upregulated the angiogenesis factors including VEGF-A and Ang-1 [83]. Curcumin may promote both neovascularization and small capillary formation in a rat model of nasal mucosal trauma [84] and improved neovascularization in diabetic model of streptozotocin and gene induced [85].

Meanwhile, neovascularization represents an important part in wound healing; angiogenesis could affect the whole process of wound healing from the very beginning after skin injury until the end of the wound remodeling [86]; curcumin has been proven as an effective natural product in wound healing, which is used as a household therapy in Indian subcontinent for management of skin diseases, wound, insect bites, and other inflammatory diseases from ancient time; relevant researches have been done to explore its proangiogenesis effect in wound healing; anti-inflammatory activity may be the main mechanism by which curcumin improves wound healing [87]. Curcumin has been shown to be a promising proangiogenesis agent in wound healing by inducing TGF-beta, which could induce both angiogenesis and accumulation of extracellular matrix through the entire remodeling phase of wound repair [88]. Ken V and his colleagues found that curcumin can enhance the neo vasculogenesis and accelerate the wound healing in diabetic rats by increasing the expressions of various factors, for example, VEGF and TGF-beta1, leading to well-formed blood vessels with increased microvessel density, which indicated that curcumin could promote angiogenesis [89]. The* in vivo* effects of curcumin on wound healing in rats and guinea pigs has been studied showing that extensive neovascularization and molecular biology analysis also showed an increase in the mRNA transcripts of TGF-beta1 in curcumin-treated wounds [90]. These results revealed the proangiogenesis effect of curcumin.

Expect for curcumin itself, ethosomal curcumin also showed the effect of promoting neovascularization in the second degree burns in rat [91] and curcumin cross-linked collagen aerogels also possesses proangiogenesis efficacy [92]. Oil of* Curcuma longa* also showed significant proangiogenesis activity [93]. All together are showing the effect of curcumin on promoting angiogenesis by regulating proangiogenesis factors.

### 5.3. Dual Effects of Curcumin on Angiogenesis

**Figure 4:**
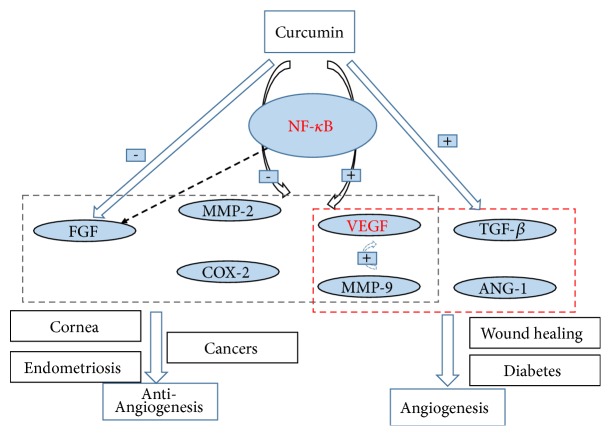
Possible mechanism on dual effect of curcumin on angiogenesis.

As discussed before, it is found that curcumin shows a dual effect on angiogenesis (Figure 4); studies have confirmed this idea that curcumin shows different effects in different microenvironment. To be specific, when cells are in a microenvironment that lack of exogenous stimuli and exposed to growth factors such as FGF, curcumin may present an antiangiogenesis effect, while proangiogenesis effect of curcumin is mediated through VEGF and PI3K-Akt pathway in different microenvironment [94], which explained the underlying mechanism of opposite effect of curcumin on angiogenesis. Meanwhile,* in vitro* study showed that curcumin reveals opposite angiogenesis effects on human umbilical vein endothelial cells and chicken chorioallantoic membrane as a function of dose [95]. Besides, the dosage of curcumin may be another factor to explain why curcumin possesses both proangiogenesis and antiangiogenesis activity; curcumin could be taken to treat different medical conditions based on the dosage used to show either proangiogenesis or antiangiogenesis effect [82]. Proangiogenesis effects of curcumin were observed at a lower dose while antiangiogenesis effects were found at higher doses [19]. It was discovered that curcumin showed a proangiogenesis effect at a low dose (20 mg/kg/day) [96], while inhibited tumor progression at a high dose (100 to 300 mg/kg/day) in C57BL/6 mice [97], indicating that the dual effect of curcumin is dose-meditated.

The inhibition of angiogenesis differentiation by curcumin depends on the serum concentration present in the incubation medium. The effective concentration required to inhibit angiogenesis in the presence of 10% serum is much higher than that required to do so in the presence of 2% serum [98].

## 6. Discussion

As reviewed in this article, it can be seen that curcumin has dual effects on angiogenesis; curcumin can not only inhibit angiogenesis in tumor and other pathological conditions, but also promote angiogenesis in fresh wound and diabetic rats, which has broadened the clinical use, pharmaceutical significance, and therapeutic applicability of curcumin, providing a new research direction on curcumin.

Even though the dual effect of curcumin has been found during years of hard work, the molecular mechanism of its dual effects has been studied in some details but the underlying mechanism of this association between angiogenesis and the dual effect of curcumin is still not crystal clear. Some researches proposed the idea that the dual effect of curcumin is dose-based or depending on the microenvironment, there is other hypothesis that chemistry structure of curcumin may contribute to it, but no solid conclusion has been drawn; further researches based on chemical genomics approach and other methods are still needed.

Such bidirectional actions of curcumin are not uncommon. Curcumin has radioprotective effects on normal tissues and radiosensitization of tumor cells [99–101], which is regulated via NF-*κ*B pathway. While curcumin shows a two-way prooxidant and antioxidant effects regulated by concentration [102–104], these findings indicated that bidirectional actions of curcumin are not uncommon and mechanisms of these bidirectional actions are different. In this way, the pro- and antiangiogenesis effects of curcumin are closely related to the concentration, and NF-*κ*B pathway plays an important role as well, indicating that the underlying mechanisms of bidirectional actions of curcumin on angiogenesis are more complicated.

There is study which showed microRNAs as a part of cellular communication [105]. Research has found that curcumin alone upregulated expression of miR-122 and downregulated miR-221 expression; in the same time, curcumin affected microvessel count, expression of angiogenesis, and microRNAs [106]. In addition, another study showed that curcumin may exert its antitumor effects via inhibiting angiogenesis through modulation of VEGF signal regulatory miRNAs [107]. As for proangiogenesis effects of curcumin, curcumin had an active role in nondiabetic peripheral arterial disease by improving angiogenesis, which may be partially achieved by promoting miR-93 expression [108]. All these findings indicated that micro-RNAs may be the principal regulator of curcumin insides the cells.

Recent researches focus mostly on the unilateral role of curcumin on angiogenesis; studies focused on antiangiogenesis effect of curcumin showed that curcumin has great potential in anticancer treatment, as a natural product with great safety and low toxicity; more preclinical and clinical studies should be conducted to dig the anticancer effect of curcumin through inhibiting angiogenesis and promote new drug research and development. Novel delivery systems of curcumin have been investigated extensively to improve the bioavailability* in vitro* and* in vivo*, and curcumin has been used in traditional medicine for many years with accurate effects and has tremendous potential in treating difficult disease; it still needs deeper researches, both evidence-based preclinical and clinical researches for evaluating its pharmaceutical potentialities and better understanding of its pharmacological mechanisms, and puts studies of curcumin translate from lab to clinical. However there are limitations to this research; there are still many deficiencies in the thesis. It is our hope that more people see the value in this natural compound and provide evidence needed to help prove its mechanisms.

## Data Availability

Readers can access the data underlying the findings of the study by contacting authors via e-mails.

## Abbreviations

bFGF: Basic fibroblast growth factor
COX-2: Cyclooxygenase-2
EGF: Epidermal growth factor
EPCs: Endothelial progenitor cells
FGF: Fibroblast growth factors
HUVEC: Human umbilical vein endothelial cells
MMPs: Matrix metalloproteinases
MCD: Mast cell density
NF-*κ*B: Nuclear factor-kappaB
TNF: Tumor necrosis factor
TGF: Transforming growth factors
VEGF: Vascular endothelial growth factor.
